# Increasing Tau 4R Tau Levels Exacerbates Hippocampal Tau Hyperphosphorylation in the hTau Model of Tauopathy but Also Tau Dephosphorylation Following Acute Systemic Inflammation

**DOI:** 10.3389/fimmu.2020.00293

**Published:** 2020-03-05

**Authors:** Matthew R. Barron, Jane Gartlon, Lee A. Dawson, Peter J. Atkinson, Marie-Christine Pardon

**Affiliations:** ^1^School of Life Sciences, Division of Physiology, Pharmacology and Neuroscience, Medical School, Queens Medical Centre, University of Nottingham, Nottingham, United Kingdom; ^2^EMEA Knowledge Centre, Eisai Ltd., Hatfield, United Kingdom; ^3^Cerevance Ltd., Cambridge, United Kingdom

**Keywords:** inflammation, lipopolysaccharide, Alzheimer's disease, mouse model, tauopathies, tau isoform imbalance

## Abstract

Inflammation is considered a mechanistic driver of Alzheimer's disease, thought to increase tau phosphorylation, the first step to the formation of neurofibrillary tangles (NFTs). To further understand how inflammation impacts the development of tau pathology, we used (hTau) mice, which express all six, non-mutated, human tau isoforms, but with an altered ratio of tau isoforms favoring 3R tau due to the concomitant loss of murine tau (mTau) that is predominantly 4R. Such an imbalance pattern has been related to susceptibility to NFTs formation, but whether or not this also affects susceptibility to systemic inflammation and related changes in tau phosphorylation is not known. To reduce the predominance of 3R tau by increasing 4R tau availability, we bred hTau mice on a heterozygous mTau background and compared the impact of systemic inflammation induced by lipopolysaccharide (LPS) in hTau mice hetero- or homozygous mTau knockout. Three-month-old male wild-type (Wt), mTau^+/−^, mTau^−/−^, hTau/mTau^+/−^, and hTau/mTau^−/−^ mice were administered 100, 250, or 330 μg/kg of LPS or its vehicle phosphate buffer saline (PBS) [intravenously (*i.v*.), *n* = 8–9/group]. Sickness behavior, reflected by behavioral suppression in the spontaneous alternation task, hippocampal tau phosphorylation, measured by western immunoblotting, and circulating cytokine levels were quantified 4 h after LPS administration. The persistence of the LPS effects (250 μg/kg) on these measures, and food burrowing behavior, was assessed at 24 h post-inoculation in Wt, mTau^+/−^, and hTau/mTau^+/−^ mice (*n* = 9–10/group). In the absence of immune stimulation, increasing 4R tau levels in hTau/mTau^+/−^ exacerbated pS202 and pS396/404 tau phosphorylation, without altering total tau levels or worsening early behavioral perturbations characteristic of hTau/mTau^−/−^ mice. We also show for the first time that modulating 4R tau levels in hTau mice affects the response to systemic inflammation. Behavior was suppressed in all genotypes 4 h following LPS administration, but hTau/mTau^+/−^ exhibited more severe sickness behavior at the 100 μg/kg dose and a milder behavioral and cytokine response than hTau/mTau^−/−^ mice at the 330 μg/kg dose. All LPS doses decreased tau phosphorylation at both epitopes in hTau/mTau^+/−^ mice, but pS202 levels were selectively reduced at the 100 μg/kg dose in hTau/mTau^−/−^ mice. Behavioral suppression and decreased tau phosphorylation persisted at 24 h following LPS administration in hTau/mTau^+/−^ mice.

## Introduction

Alzheimer's disease (AD), the most prevalent senile dementia, is characterized by the presence of amyloid beta (Aβ) plaques and neurofibrillary tangles (NFTs) caused by hyperphosphorylation and aggregation of the microtubule associated protein tau. Their progressive accumulation is associated with cognitive deterioration, brain atrophy, and neuroinflammation, but tau pathology correlates better with disease progression than Aβ deposition (1, 2). The adult human brain expresses six main tau isoforms, which can be categorized as 3R or 4R tau based on whether they contain three or four carboxy-terminal repeat domains (3). In the healthy brain, the 3R and 4R isoforms are found in equal amounts (4). However, alterations in the 4R:3R tau isoform ratio, often caused by mutations in the tau gene, trigger pathological tau aggregation and NFT formation (5). Resulting tauopathies are classified in three groups according to the predominant species of tau that accumulate: 4R tauopathies (e.g., progressive supranuclear palsy, corticobasal degeneration), 3R tauopathies (e.g., Pick's disease), and 3R+4R tauopathies (e.g., AD) (6, 7); but in AD, the trigger of tau pathology is not genetic. NFTs of AD patients express both isoforms, and a number of studies reported a 4R:3R ratio of ~1, comparable to control brains (4, 8–10), suggesting that tau aggregation in AD is not due to isoform imbalance. Nevertheless, changes in the 4R:3R tau isoform ratio in AD brains have also been reported with borderline to significant increases (11–15) or decreases (16, 17) compared to controls. Importantly, this ratio is subject to significant variability between AD samples. To clarify these discrepancies, more detailed analyses have looked at the differential accumulation of 3R and 4R tau in the human brain in relation to AD severity (17, 18), to the brain areas investigated (11, 13, 16), and to region-specific differences in susceptibility to NFT formation within the brain (16), as well as in the NFTs of AD patients where three distinct isoform patterns of tau coexist, containing either 3R tau, 4R tau or both. Shifts in patterns between neurons and brain regions were found to be associated with the stage of NFT pathology (19–22). Overall, these detailed studies suggest that the predominance of 3R tau over 4R tau increases with the progression of NFT pathology.

The causes of sporadic AD are far from being fully understood, but systemic inflammation is now seen as a key mechanistic driver of AD pathogenesis. Genes associated with immune cell function and susceptibility to inflammatory diseases (23, 24) as well as systemic inflammatory conditions such as rheumatoid arthritis, Type 2 diabetes, and obesity (25–27) increase the risk of developing AD. Circulating levels of systemic inflammatory mediators such as interleukin 1β (IL-1β) and tumor necrosis factor alpha (TNFα) were found chronically upregulated in AD (28). Post-mortem examinations of AD brains show a sustained activation of immune cells, particularly surrounding NFTs (29). Moreover, systemic infections accelerate the rate of cognitive decline in AD patients, even after the resolution of the initial inflammatory insult (30, 31). One potential mechanism mediating the impact of systemic inflammation on AD progression is through the exacerbation of tau pathology. Kinases involved in tau phosphorylation, such as cyclin-dependent kinase-5 and mitogen activated kinases, can be induced by inflammatory signaling cascades (32–34). A number of preclinical mouse studies using lipopolysaccharide (LPS) to mimic gram-negative bacterial infection reported exacerbation of tau pathology. For instance, a systemic LPS dose of 10 mg/kg was found to increase tau phosphorylation in wild-type (Wt) mice, whereas exacerbation of tau hyperphosphorylation was seen with 10 times less LPS (1 mg/kg) in hTau mice (35), a mouse line expressing exclusively the six human tau isoforms (36). At 0.5 mg/kg, LPS was also found to increase tau phosphorylation and aggregation in the 3xTg model, which expresses both tau and Aβ pathology (37, 38). Yet, these preclinical studies have used high LPS doses more relevant to sepsis than to the chronic low-grade inflammation associated with AD (39).

LPS is an endotoxin produced in the gut and a potent stimulator of the innate immune system. At low doses able to induce physiologically relevant inflammation, LPS does not enter the brain in the absence of blood–brain barrier dysfunction (40, 41). Recent reports indicate that circulating concentrations and brain penetration of LPS increase in AD patients, in which LPS co-localizes with Aβ plaques and neurons (42–44). This led to the hypothesis that endogenous LPS accumulation could play a critical role in the pathophysiology of sporadic AD (45). There is, therefore, a need to better understand the mechanisms whereby systemic LPS affects tau pathology. Here we focused on the impact on tau phosphorylation at the onset of systemic inflammation with a low dose of LPS, using the hTau model. This mouse line is thought to be the most relevant tau model for AD. It involves the expression of all six, non-mutated, human tau isoforms, but the altered ratio of tau isoforms favoring 3R tau due to the concomitant loss of murine tau (mTau) that is predominantly 4R is thought to underlie pathological changes in this model (36). In favor of this hypothesis, restoring tau isoform imbalance to a 4R:3R ratio of ~1 by increasing 4R tau levels was found to alleviate tau pathology and cognitive deficits in these mice (46). On the other hand, increasing 4R tau and decreasing 3R tau expression without altering total tau levels was found to worsen tau pathology and associated behavioral deficits in hTau mice (47). And although removal of mTau was found necessary for the tau pathology to occur in hTau mice (36), we have shown previously that heterozygous deletion of mTau is sufficient for robust behavioral deficits and tau phosphorylation to occur in this model (48). While these data show that direct modulation of the 4R:3R ratio in the brain of hTau mice alters tau pathology, whether or not this also affects susceptibility to systemic inflammation and LPS-induced changes in tau phosphorylation is currently not known.

To address this question, we bred hTau mice on a heterozygous mTau background (hTau/mTau^+/−^) to reduce the predominance of 3R tau by increasing availability of 4R tau. We predicted that this would alleviate behavioral and pathological changes in hTau mice and protect them against exacerbation of tau pathology after mild systemic inflammation with LPS. Here, we first report that increased 4R tau levels in hTau/mTau^+/−^ mice exacerbate tau hyperphosphorylation compared to hTau mice on a full mTau knockout background (hTau/mTau^−/−^). Since systemic inflammation is expected to be an early event in the pathogenesis of AD, we used 3-month-old mice, corresponding to the onset of behavioral changes in these models (48, 49). We also report that increasing 4R tau availability was associated with a rapid reduction in tau phosphorylation in the hippocampus, seen 4 h after inoculation with low doses of LPS, and persisting for at least 24 h.

## Methods

### Animals

Breeding stock of hTau mice on a C57/BL6J background and their mTau^−/−^ control [STOCK *Mapt*^*tm*1(*EGFP*)*Klt*^ Tg(MAPT)8cPdav/J] were originally purchased from The Jackson Laboratory (Bar Harbor, ME, USA, stock #005491). They were then crossed with Wt C57/BL6J mice to produce mTau^+/−^ and hTau/mTau^+/−^ breeders in the University of Nottingham Biomedical Service Unit. This enabled the production of all experimental animals from five genotypes as littermates: Wt, mTau^+/−^, mTau^−/−^, hTau/mTau^+/−^, and hTau/mTau^−/−^. Previous studies investigating the impact of modulation of the 4R:3R tau isoform ratio on tau pathology have used either males (46) or both males and females but without testing for sex differences (47), although sex differences in disease progression have been reported in hTau mice (50). As we did not have an a priori hypothesis on sex differences in the impact of 4R tau availability on immune responses, we only used male mice for this study. They were maintained under standard husbandry conditions on a 12/12 h light cycle, with lights on at 07:00 h and room temperature, relative humidity, and air exchange automatically controlled. Mice were kept grouped housed in individually ventilated cages (IVCs) with *ad libitum* access to food and water, and provided with a play tube and nesting material. All animal procedures were carried out in accordance with the UK Animals Scientific Procedures Act under project license 40/3601, approved by the University of Nottingham Animal Welfare and Ethical Review Board and reported according to the ARRIVE guidelines (51). All analyses were performed blind.

### Drug Treatment

LPS (*Escherichia coli* serotype Sigma0111:B4, Sigma Aldrich) was dissolved in phosphate buffer saline (PBS, Sigma Aldrich) and stored in aliquots at −20° until use. The day of the experiment, mice were injected intravenously (i.v.) in the dorsal tail vein with 100, 250, or 330 μg/kg of LPS or its vehicle PBS at a volume of 1 μl/g of body weight, as previously described (52). These doses were selected to mimic a low-grade systemic inflammation as seen in AD patients. They were previously shown to induce a mild transient sickness syndrome associated with the expression of pro-inflammatory mediators, reversible by anti-inflammatory drugs (53, 54).

### Study Design

#### Comparison of Tau Pathology in 9-Month-Old hTau/mTau^+/−^ and hTau/mTau^−/−^ Mice

To assess the impact of increasing 4R tau availability on tau aggregation, we used 9-month-old naïve male Wt, mTau^+/−^, mTau^−/−^, hTau/mTau^+/−^, and hTau/mTau^−/−^ mice (*n* = 4/group), an age when sarkosyl-insoluble tau can be expected in the brain of hTau mice (36). Animals were sacrificed by cervical dislocation; one hemisphere was snap-frozen for subsequent assessment of sarkosyl-insoluble tau, while the other hemisphere was fixed in 4% paraformaldehyde (PFA) for 6 h at room temperature and paraffin embedded for immunohistological analyses.

#### Dose-Dependent Effects of LPS on Sickness Symptoms, Circulating Cytokine Levels, and Tau Phosphorylation in hTau/mTau^+/−^ and hTau/mTau^−/−^ Mice at Onset of Systemic Inflammation

**Figure 1:**
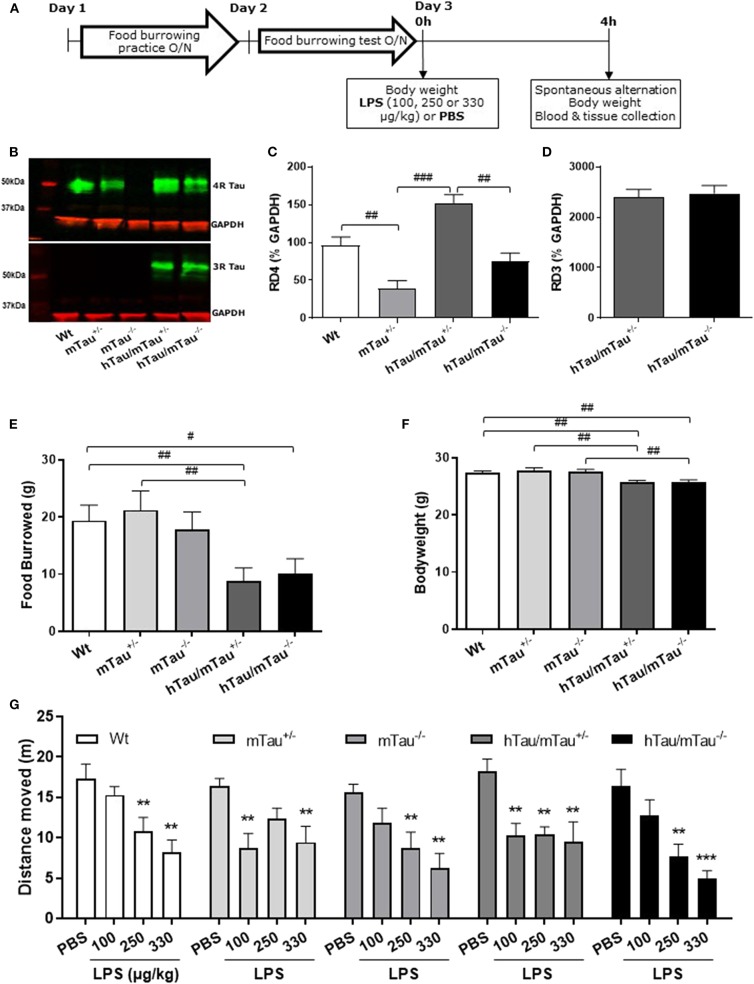
Increasing 4R tau levels in hTau mice modulates lipopolysaccharide (LPS)-induced behavioral suppression at 4 h post-injection. Data are expressed as means ± standard error of the mean (SEM). **(A)** Timeline of the experiment. Three-month-old male wild-type Wt, mTau^+/−^, mTau^−/−^, hTau/mTau^+/−^, and hTau/mTau^−/−^ mice were subjected to baseline assessment of food burrowing performance prior to receiving a tail vein injection of LPS (100, 250, or 330 μg/kg) or its vehicle (phosphate buffer saline, PBS; *n* = 8–9/group). Spatial working memory and LPS-induced sickness effects were tested at 4 h post-injection in the spontaneous alternation test, the latter by measuring locomotor activity in the Y-maze, prior to blood and tissue collection. **(B–D)** Hippocampal tau isoform levels were determined by western immunoblotting **(B)**. Breeding hTau mice on a heterozygous murine tau (mTau) knockout background significantly increased hippocampal 4R tau levels **(C)**, without altering 3R tau levels **(D)**. Since LPS had no statistically significant effects on tau isoform levels, which are genetically determined, treatment groups were subsequently pooled for each genotype, for the sake of clarity. *n* = 34–36 per genotype. Pairwise comparisons: ^#^*p* < 0.05; ^##^*p* < 0.01; ^###^*p* < 0.0001. **(E–F)** In the absence of immune stimulation, food burrowing behavior was impaired **(E)** and body weight was reduced **(F)** in hTau mice, regardless of the mTau background. *n* = 34–36 per genotype. Pairwise comparisons: ^#^*p* < 0.05; ^##^*p* < 0.01; ^###^*p* < 0.0001. **(G)** Four hours after inoculation with systemic LPS, exploratory drive in the spontaneous alternation test, assessed through the distance moved, was suppressed in all genotypes, but in a dose-dependent manner. hTau/mTau^+/−^ mice and their heterozygous mTau knockout (mTau^+/−^) littermates were more susceptible to the lowest LPS dose, but, unlike what was seen in the other three genotypes, they did not show further behavioral suppression with increasing LPS doses. *n* = 8–9/group. Pairwise comparisons: **p < 0.01; ***p < 0.0001 vs. PBS.

Three-month-old male Wt, mTau^+/−^, mTau^−/−^, hTau/mTau^+/−^, and hTau/mTau^−/−^ mice (*n* = 8/9/group) were randomly allocated to the PBS or LPS groups. The timeline of the experiment is shown Figure 1A. Mice were subjected to baseline behavioral assessment prior to being challenged with PBS or LPS. On Day 1, they were trained to burrow food overnight in groups, and on Day 2, they underwent baseline food burrowing testing for 4 h, singly housed. On Day 3, mice were challenged with LPS (100, 250, or 330 μg/kg *i.v*.) or PBS (1 μl/g of body weight, *i.v*.). Post-treatment spatial working memory performance was assessed 4 h later in the spontaneous alternation test, and locomotor activity was recorded as an indicator of LPS-induced sickness effects. Mice were then immediately sacrificed by cervical dislocation. Trunk blood was collected for quantification of cytokine levels, in order to determine whether 4R availability impacts upon the systemic response of hTau mice to LPS. The brains were removed, and the hippocampi were dissected from one hemisphere and snap-frozen for subsequent assessment of total and phosphorylated tau levels by western immunoblotting. For six animals per group, the other hemisphere was snap-frozen for analysis of sarkosyl-insoluble tau species, while for the remaining two to four animals, one hemisphere was fixed in PFA for 6 h and paraffin embedded for immunohistological analyses of tau.

#### Persistence of LPS Effects on Systemic Inflammation, Behavioral Sickness, and Tau Phosphorylation at 24 h Post-Inoculation

We determined the persistence of LPS-induced alterations in tau pathology in 3-month-old male Wt, mTau^+/−^, and hTau/mTau^+/−^ mice 24 h following LPS administration, as hTau/mTau^−/−^ mice were less affected by the immune challenge. The timeline of the experiment is illustrated **Figure 5A**. Mice first underwent a grouped food burrowing practice (Days 1–4), followed, on Day 5, by baseline assessment of food burrowing performance overnight and spontaneous alternation behavior. On Day 8, mice were administered with either PBS or 250 μg/kg LPS (*i.v., n* = 9–10/group), as this was the most effective dose, and assessed for LPS-induced sickness in the food burrowing test overnight and in the spontaneous alternation test 24 h after the injection. They were then culled by cervical dislocation; trunk blood and brain tissue were collected as described above.

### Behavioral Assessment

#### Food Burrowing

Food burrowing is a species-specific behavior largely dependent on the integrity of the hippocampus (55), which is suppressed in response to systemic inflammation (56, 57) and is sensitive to the progression of tau pathology in hTau mice (49). The protocol was adapted from one previously described (49). For the overnight practice of food burrowing in groups, a jar containing 50 g of food pellets broken into small pieces was added to the home cage between 17:00 and 18:00 and removed between 09:00 and 10:00 the next day. To assess food burrowing performance, singly housed animals were provided with a jar containing 50 g of food pellets overnight, environmental enrichment, and *ad libitum* access to food and water. The amount of food displaced from the jar was recorded, expressed as a percentage of the 50 g provided, and used as a measure of food burrowing performance.

#### Spontaneous Alternation

Spontaneous alternation was used as previously described (49) to assess spatial working memory and exploratory drive. The latter is suppressed in response to LPS-induced sickness and is a potential confounding factor for the assessment of cognitive effects (58). The Y-maze comprised three transparent Plexiglas arms (41.5 cm in length and 6 cm in width surrounded by 15 cm high transparent Perspex walls) at a 120° angle from each other each. The start point (6 × 7.5 cm) was located in the center of the maze, and the mice were allowed to freely explore the three arms over 5 min. The total distance moved in the whole maze was automatically tracked using the Ethovision software (v.10, Noldus, Wageningen, Netherlands) and used as an indication of exploratory drive. A correct alternation entailed a mouse entering three different arms in a row. The number of alternations was recorded manually and expressed as a percentage of alternations to estimate spatial working memory performance.

### Assessment of Tau Pathology

#### Western Immunoblotting

To assess tau pathology, western immunoblotting of tau species and protein phosphatase 2A catalytic (PP2Ac) activity was carried out on hippocampal tissue. Briefly, tissue was homogenized in 5 × radioimmunoprecipitation assay (RIPA) buffer [50 mM Tris-HCl (Sigma, #T5941), 0.1% Triton X-100 (Sigma, X100), 0.25% Na-deoxycholate (Sigma, #D6750), 150 mM NaCl (Sigma, #S3014), 1 mM Ethylenediaminetetraacetic acid (EDTA), (Sigma, #EDS)] containing phosphatase 1 mM Na_3_VO_4_ (Sigma, #S6508), 1 mM NaF (Sigma, #S7920), and complete protease inhibitor (Roche, #11697498001). Samples were then centrifuged at 20,000 Relative Centrifugal Force (RCF) for 20 min and the resulting supernatant, corresponding to the cytosolic fraction, was collected. Total protein levels were measured using the bicinchoninic acid (BCA) assay (Novagen, #71285) and standardized in RIPA buffer. Samples were diluted 1:2 in Laemmli buffer (Sigma, #3401) and heated at 100°C for 5 min to denature. For determination of PP2A activity, prior to heating at 100°C, samples were pre-treated with 0.1M NaOH for 30 min at 37°C followed by neutralization with HCl to completely demethylate samples. The PP2Ac subunit is regulated by methylation of its 309L residue, which induces its activation. The PP2Ac antibody 05-421 (Merck) binds PP2Ac at this position but cannot if the molecule is methylated, binding only inactive PP2A (59).

Following denaturation at 100°C, 15 μg of protein was resolved on 7.5% Criterion TGX gels (BioRad Laboratories) and transferred to Amersham Protran 0.45NC nitrocellulose membranes (GE Healthcare, #10600002). Membranes were blocked with 5% milk in Tris buffered saline (TBST; 0.1% tween 20) for 1 h at room temperature and probed overnight at 4°C in blocking buffer with anti-rabbit GAPDH (1:40;000, G8795; Sigma Aldrich) as a loading control; anti-mouse Tau46 (1:500, T9450; Sigma Aldrich) for total tau; anti-mouse CP13 (1:500, pS202) and anti-mouse PHF1 (1:500, pS396/404), both generously gifted by Prof. Peter Davies (NY, USA), for early- and late-pathological-stage tau phosphorylation, respectively; anti-mouse RD3 (1:1,000, 05-803, Millipore) and anti-mouse RD4 (1:500, 05-804, Millipore), for 3R and 4R tau isoforms, respectively; and anti-mouse PP2A (1:5,000, 05-421, Millipore) targeting PP2Ac. After washing with TBST, membranes were incubated with infrared fluorescent secondary antibodies IRDye 680RD Goat anti-Rabbit (1:15,000, 926-68071; LI-COR) and IRDye 800CW Goat anti-Mouse (1:15,000, 926-32210; LI-COR) in blocking buffer for 1 h at room temperature. Blots were scanned and analyzed using the Odyssey infrared imaging system (LI-COR Biosciences). Band intensities were normalized to GAPDH. In the dose–response experiment, a ratio between either pS202 or pS396/404 and tau46 was calculated to provide a ratio of phosphorylated: total tau. For PP2A activity, the ratio between non-NaOH and NaOH treated samples was calculated to provide a ratio of inactive: total PP2Ac.

#### Tau Aggregation

Aggregated tau was assessed by isolating insoluble tau *via* a sarkosyl extraction method validated in house using rtg4510 mice. Hemibrains were homogenized in 11 × (w/v) ice-cold homogenization buffer [high-salt and -sucrose solution: 50 mM Tris-HCl (pH 7.5, Sigma, #T2319), 10% sucrose (Fisher, #57-50-1), 5 mM EDTA (Sigma, T9285), 800 mM NaCl (Sigma, #S1679), okadaic acid (Merck, #459620), α2-macroglobulin (Sigma, #10602442001), and Halt protease and phosphatase inhibitors (Thermo Scientific, #78440)]. The homogenates were then spun at 6,000G for 15 min at 4°C and the supernatant saved. The pellet was re-suspended in 5 × (w/v) homogenization buffer and mixed with the previously collected supernatant, producing the total homogenate fraction. Total protein concentrations were then determined with the BCA assay to ensure that sarkosyl extraction of insoluble tau was carried out using an equal amount of proteins in all mice. Total homogenates were then diluted to 5 μg/μl in homogenization buffer and centrifuged at 14,000G for 15 min at 4°C. The total homogenate supernatant was collected (S1) and the pellet (P1) discarded. The S1 fraction was incubated in 1% sarkosyl for 1 h at room temperature and centrifuged at 160,000G for 30 min at 4°C, and the resulting sarkosyl-soluble supernatant (S2) was collected. A wash spin was conducted by adding 2 ml of homogenization buffer to the pellet and centrifuging again at 160,000G for 30 min. The pellet was then re-suspended in a solubilization buffer (50 mM Tris-HCl, 2.3% SDS, 1 mM EDTA, and Halt protease and phosphatase inhibitors), providing the sarkosyl-insoluble fraction (P2). Fractions were stored at −80°C until use for western immunoblotting of S1 and P1 fractions with anti-mouse Tau46 for total tau and anti-mouse PHF1 for late-pathological-stage tau phosphorylation, as described above, but with sarkosyl-insoluble P2 fractions being resolved on 4–12% gels.

#### Immunohistochemical Assessment of Tau Pathology

Serial coronal sections 7 μm thick were cut throughout the hippocampus using a microtome (Microtome Slee Cut 4060), mounted on APES (3-aminopropyltriethoxysilane)-coated slides, and dried overnight at 40°C. For immunostaining of tau species, antigen retrieval was performed in boiling sodium citrate buffer (10 mM tri-sodium citrate, pH 6.0, Fischer #S/3320/53) for 20 min. Endogenous peroxidases were then quenched in 3% H_2_O_2_ in methanol for 5 min. Brain slices were blocked for 1 h at room temperature (RT) using mouse-on-mouse Immunoglobulin G (IgG) blocking reagent (Vector, #MKB-2213). Primary antibody incubation with anti-mouse Tau46 (1:400), CP13 (1:25, pS202), or PHF1 (1:25, pS396/404) in blocking buffer was carried out overnight at 4°C. Secondary antibody incubation consisted of biotinylated horse anti-mouse secondary antibody (1:200 in blocking buffer, Vector Laboratories, #BMK-2202), applied for 1 h at RT. Tissue was then washed, exposed to ABC-HRP (Vectastain Elite ABC Kit R.T.U, Vector Labs, cat. nr. PK-7100) and labeled with DAB (3,3′-Diaminobenzidine) peroxidase substrate (Vector Labs cat. SK-4100) according to manufacturer's instructions, and finally, counterstained using hematoxylin. Digital focused photo-scanning images were then acquired using a Hamamatsu NanoZoomer-XR 2.0-RS C10730 digital scanning system with time delay integration (TDI) camera technology (Hamamatsu Photonics K.K. Systems, Japan) at 20 × magnification and visualized using NDP.view2 (NanoZoomer Digital Photography). Tau localization was assessed qualitatively.

### Multiplex

Plasma levels of IL-1β, IL-6, IL-10, interferon gamma (IFN-γ), and TNFα were determined using the Bio-Plex ProTM Mouse Cytokine 23-Plex, Group I assay, and Bio-Plex array reader, and analyzed using the Bio-Plex Manager Software (Bio-Rad Laboratories, Berkeley, CA, USA) according to the manufacturer's instructions. The cytokine panel was designed to provide a measure of key cytokines known to respond to LPS and to play a role in AD.

### Data Analysis

Data are presented as mean ± SEM (standard error of the mean). ANOVAs and planned comparisons, decided a priori, were carried out using InVivoStat (60) to compare relevant experimental groups. The significance of relevant pairwise comparisons was determined by using ANOVAs with *post hoc* Fisher's least significant difference (LSD), rather than *post hoc* tests with correction for multiple comparisons, because the whole experimental design was planned to test a few specific hypotheses that are reflected by the small subset of chosen planned comparisons. Therefore, the risk of false positives (Type 1 errors) is unlikely and further reduced by using the variance estimate generated by the ANOVA models, using all animals within the experiment. This approach also has a lower chance of declaring false negative results (Type 2 errors) arising from the high number of possible comparisons. The number of arm entries was used as a covariate for the analysis of spontaneous alternation performance to control for confounding effects of LPS-induced behavioral suppression. Baseline behavioral and body weight data, as well as analysis of tau isoforms and species in hemibrains from 9-month-old mice, were subjected to one-way ANOVAs with genotype as a between factor, followed, where appropriate, by planned comparisons. The following pairwise comparisons were decided a priori (7 out of 10 possible comparisons): (i) Wt vs. each of the four genetically altered groups to test for genotype differences; (ii) hTau/mTau^+/−^ and hTau/mTau^−/−^ vs. their respective mTau control, to test for human tau expression within each mTau genotype; and (iii) hTau/mTau^+/−^ vs. hTau/mTau^−/−^ to test for the impact of 4R tau availability on the hTau phenotype. The effects of LPS on tau isoforms and pathology as well as cytokine data were assessed by two-way ANOVAs with genotype and either doses (4 h time point) or treatment (24 h time point) as between-subject factors, followed, where appropriate, by planned comparisons. For analyses of tau species and 4R tau, mTau^−/−^ mice were excluded, as they do not express tau. Analyses of 3R tau were only conducted in the two hTau models, as non-genetically altered mice do not express 3R tau. To account for gel-to-gel variability in western immunoblotting data, a randomized block analysis of variance was conducted, followed, when appropriate, by planned comparisons. Data for phosphorylated:total tau ratios were log transformed to normalize the distribution. The following pairwise comparisons were decided a priori (39 out of 190 possible comparisons for the 4 h time point and 6 out of 15 possible comparisons for the 24 h time point): (i) PBS-treated Wt vs. each of the four genetically altered groups to test for genotype differences in the absence of immune stimulation; (ii) PBS-treated hTau/mTau^+/−^ and, if appropriate, hTau/mTau^−/−^, vs. their respective PBS-treated mTau control, to test for the effect on human tau expression within each mTau genotype; (iii) PBS- *vs*. LPS-treated mice for each LPS dose within each genotype to test for differences caused by systemic inflammation with LPS; (iv) for the 4 h time point only, hTau/mTau^+/−^ vs. hTau/mTau^−/−^ for each LPS dose to test for the impact of 4R tau availability on the hTau phenotype in the absence and presence of immune stimulation; and (v) LPS doses within each genotype to test for dose-dependent effects. To assess the persistence of LPS-induced sickness at 24 h post-inoculation, we compared baseline and post-injection data using two-way ANOVAs with genotype and treatment as between-subject factors and repeated measures over time, followed by planned comparisons. The following pairwise comparisons were decided a priori (8 out of 66 possible comparisons): (i) Wt vs. each of the two genetically altered groups prior to injection with PBS or LPS, to test for genotype differences at baseline, and (ii) baseline vs. post-injection data within each experimental group to test for the effect of the PBS or LPS challenge and/or repeated testing. *P* ≤ 0.05 was considered statistically significant.

## Results

### Increased 4R Tau Availability in hTau/mTau^+/−^ Exacerbates Tau Hyperphosphorylation Without Worsening Behavioral Disturbances

We have previously shown that hTau mice bred on a heterozygous mTau knockout background develop behavioral and pathological features of tauopathies (48). Using hemibrains from 9-month-old mice, we first aimed to determine whether this breeding strategy impacted on tau aggregation, but neither hTau/mTau^+/−^ nor hTau/mTau^−/−^ mice exhibited aggregated tau (Supplementary Figure 1G). Next, we used this tissue for verification of the expected increase in 4R tau (Supplementary Figure 1B) without affecting 3R tau levels (Supplementary Figure 1C) in hTau/mTau^+/−^ mice. This preliminary experiment on a limited number of animals also suggested that increased 4R tau availability was associated with elevated levels of total, early-, and late-pathological-stage phosphorylated tau (Supplementary Figures 1D,E).

To address the possible impact of tau isoform ratio imbalance in the hippocampal response and sickness response to systemic inflammation, hTau mice were therefore bred on either an mTau^+/−^ or mTau^−/−^ background and dosed with PBS or LPS at 3 months of age. We first confirmed that hippocampal levels of 4R tau (Figure 1C) were significantly affected by the genotype [*F*_(3, 117)_ = 19.09, *p* < 0.0001], regardless of treatment [LPS doses: *F*_(3, 117)_ = 0.92, *p* = 0.43; genotype × LPS doses: *F*_(9, 117)_ = 0.11, *p* = 0.99]. Accordingly, hippocampal levels of 4R tau were reduced by half in mTau^+/−^ compared to Wt mice (*p* = 0.0004) and significantly higher in hTau/mTau^+/−^ than in both mTau^+/−^ (*p* < 0.0001) and hTau/mTau^−/−^ (*p* < 0.0001, Figure 1C), while 3R levels did not differ between hTau/mTau^+/−^ and hTau/mTau^−/−^ mice [*F*_(1, 48)_ = 0.23, *p* = 0.63, Figure 1D], regardless of treatment [LPS doses: *F*_(3, 48)_ = 1.23, *p* = 0.31; genotype × LPS doses: *F*_(3, 48)_ = 0.02, *p* = 0.99], confirming our preliminary findings in the whole brain of 9-month-old mice (Supplementary Figure 1). Next, we verified the presence of pathological tau features in 3-month-old mice using data from PBS-treated mice. This also indicated that increasing 4R tau levels in hTau mice were associated with elevated hippocampal tau phosphorylation at the pS202 (*p* = 0.003 vs. hTau/mTau^−/−^ mice; **Figure 3B**) and pS396/404 epitopes (*p* < 0.0001 vs. hTau/mTau^−/−^ mice; **Figure 3C**), although total tau levels were not affected (**Figure 3D**). PBS-treated hTau/mTau^+/−^ also presented with an elevated pS202:tau46 ratio compared to both PBS-treated Wt (*p* = 0.03) and mTau^−/−^ (*p* = 0.0002) mice (Supplementary Figure 4A), but the ratio of late-stage phosphorylated tau to total tau was not different between the genotypes (Supplementary Figure 4B). Tau redistribution is another pathological feature of tauopathies, whereby hyperphosphorylated tau dissociates from microtubules and relocates from the axon to the somatodendritic compartment (61). We therefore examined whether this also occurred in 3-month-old hTau/mTau^+/−^ mice using pS202 immunostaining, as a small ring of positive CP13 is expected around cell bodies upon somatodendritic tau redistribution (36). While there was mild evidence of tau in the somatodendritic compartment of both Wt and mTau^+/−^ mice, strong CP13 positive rings were observed in hTau/mTau^+/−^ and hTau/mTau^−/−^ mice (Supplementary Figure 2), indicating redistribution of phosphorylated tau and confirming our previous observation in 6-month-old hTau mice bred on a heterozygous mTau knockout (48).

We have previously shown that the food burrowing task is the most sensitive test to assess early behavioral perturbations in hTau mice bred on either an mTau^−/−^ (49) or mTau^+/−^ (48) background, and that this behavior is suppressed 4 h after inoculation with LPS in an APP/PS1 mouse model of AD and their Wt littermates (57). Here, mice were assessed for food burrowing performance prior to being challenged with PBS or LPS, and we found that the severity of the food burrowing deficit in 3 -month-old hTau mice was independent of the mTau genotype [genotype: *F*_(4, 171)_ = 3.89, *p* = 0.005; hTau/mTau^+/−^: *p* = 0.002 vs. Wt; hTau/mTau^−/−^: *p* = 0.02 vs. Wt; Figure 1E]. We also found that both hTau models weighed less than their Wt and respective mTau knockout littermates [genotype: *F*_(4, 171)_ = 6.91, *p* < 0.0001; hTau/mTau^+/−^: *p* = 0.003 vs. Wt and *p* = 0.0002 vs. mTau^+/−^; hTau/mTau^−/−^: *p* = 0.004 vs. Wt and *p* = 0.0009 vs. mTau^−/−^; Figure 1F]. Behavioral performance in the spontaneous alternation test, however, did not differ between PBS-treated mice of all five genotypes (Figure 1G for distance moved and Supplementary Figure 3A for alternation rate). Thus, while modulation of 4R tau availability in hTau mice impacts on tau phosphorylation, this is not associated with differences in early behavioral and physiological disturbances.

### 4R Tau Availability Modulates the Susceptibility to LPS-Induced Sickness in hTau Mice

To determine whether the systemic inflammation-induced sickness in hTau mice was affected by increasing 4R tau availability, locomotor activity was tracked in the spontaneous alternation task 4 h after inoculation with LPS. The distance moved in the Y-maze was dose-dependently depressed by LPS [*F*_(3, 153)_ = 28.26; *p* < 0.0001] regardless of the genotype [*F*_(4, 153)_ = 1.56, *p* = 0.018; Figure 1G], but the lowest LPS dose (100 μg/kg) was only significantly effective in inducing behavioral suppression in hTau/mTau^+/−^ mice (*p* = 0.0006 vs. PBS) and their heterozygous mTau knockout littermates (*p* = 0.001 vs. PBS) without declining further with increasing LPS doses, in contrast to other genotypes (Figure 1G). This suggests that 4R tau levels modulate the sickness response to low LPS doses. Spontaneous alternation performance, however, was not significantly altered by LPS [LPS doses: *F*_(3, 140)_ = 0.58, *p* = 0.63; genotype × LPS doses: *F*_(12, 140)_ = 1.51, *p* = 0.13; Supplementary Figure 3).

### 4R Tau Availability Limits the Cytokine Response to Higher LPS Doses in hTau Mice at 4 h Post-Inoculation

**Figure 2:**
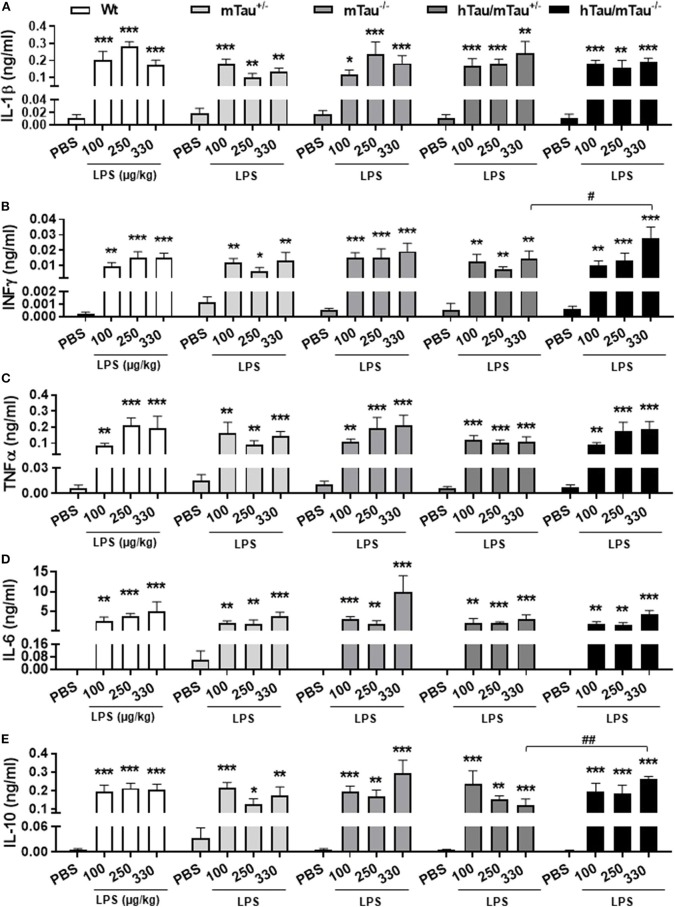
LPS-induced serum cytokines at 4 h post-injection. Three-month-old male Wt, mTau^+/−^, mTau^−/−^, hTau/mTau^+/−^, and hTau/mTau^−/−^ mice were challenged with LPS [100, 250, or 330 μg/kg, *intravenously (i.v*.)] or its vehicle PBS. Their serum was collected 4 h later, immediately after behavioral assessment, for measurement of induced levels of pro- and anti-inflammatory cytokines. At this time point, significant increases in circulating levels of the pro-inflammatory cytokines interleukin 1β (IL-1β, **A**), interferon gamma (IFN-γ, **B**) and tumor necrosis factor alpha (TNFα, **C**); of IL-6 **(D)**, which has both pro- and anti-inflammatory effects; and of the anti-inflammatory cytokine IL-10 **(E)** were observed regardless of the genotype and LPS dose. At the highest LPS dose (330 μg/kg), however, LPS-induced IFN-γ **(B)** and IL-10 **(E)** were significantly higher in hTau/mTau^−/−^ than seen in hTau/mTau^+/−^, suggesting that expression of 4R mTau in hTau mice dampens the cytokine response to stronger immune stimulations. Data are expressed as means ± SEM. *n* = 5–9/group. Pairwise comparisons: *p < 0.05, **p < 0.01, ***p < 0.0001 vs. PBS and ^#^*p* < 0.05, ^##^*p* < 0.01 vs. hTau/mTau^−/−^ mice.

To determine whether elevated 4R tau affects the extent of the cytokine response to systemic LPS administration, we quantified circulating levels of the pro-inflammatory cytokines IL-1β, IL-6, INFγ, and TNFα and prototypical anti-inflammatory cytokine IL-10. Indicating a strong pro-inflammatory response had occurred in response to all LPS doses, serum concentrations of all cytokines were significantly elevated [IL-1β: *F*_(3, 119)_ = 44.95, *p* < 0.0001, Figure 2A; INFγ: F_(3, 121)_ = 45.39, *p* < 0.0001, Figure 2B; TNFα: *F*_(3, 121)_ = 48.37, *p* < 0.0001, Figure 2C; IL-6: *F*_(3, 120)_ = 47.40, *p* < 0.0001, Figure 2D; and IL-10: *F*_(3, 121)_ = 45.07, *p* < 0.0001, Figure 2E], with little variations between doses within each genotype. Four hours after inoculation with the 330 μg/kg dose, however, LPS-induced secretion of INFγ and IL-10 were significantly lower in hTau/mTau^+/−^ than in hTau/mTau^−/−^ mice (Figures 2B,E, respectively), suggesting that 4R tau availability modulates the cytokine response to systemic immune stimulation in a dose-dependent manner.

### 4R Tau Availability Exacerbates the Rapid Tau Dephosphorylation Induced by LPS in hTau Mice

**Figure 3:**
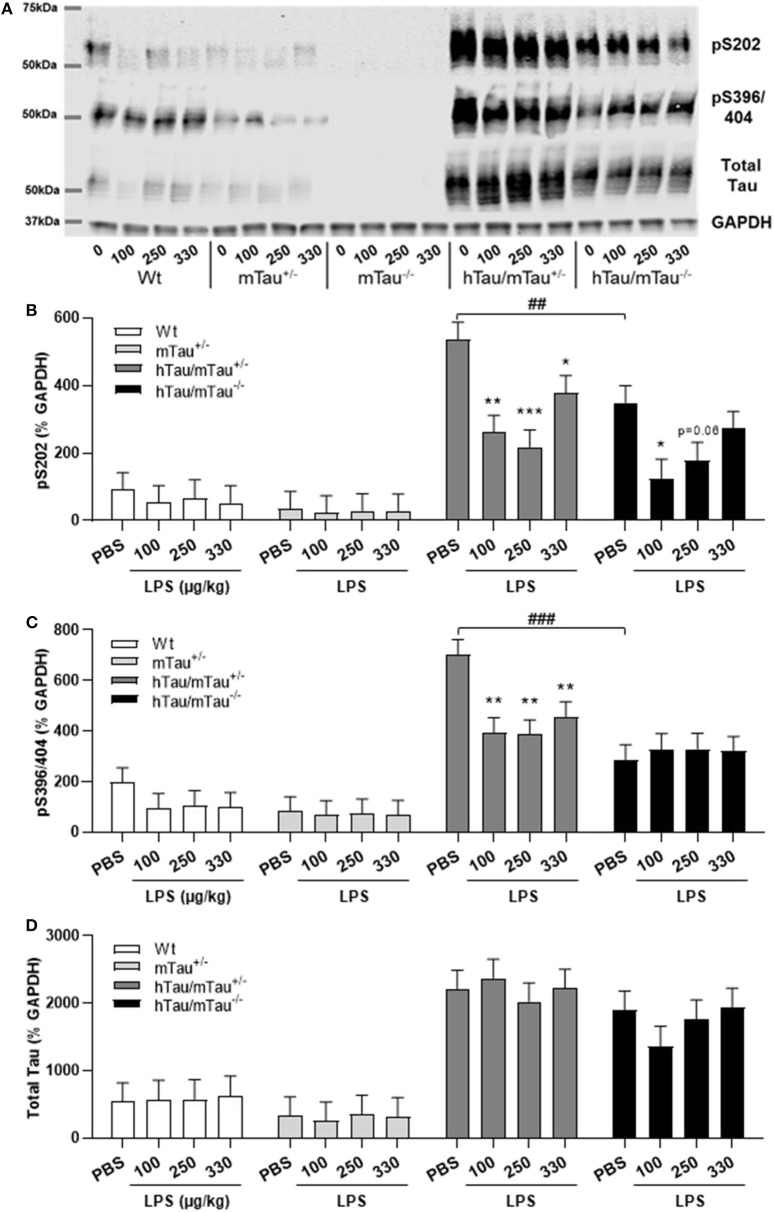
LPS-induced tau dephosphorylation at 4 h post-injection. Three-month-old male Wt, mTau^+/−^, mTau^−/−^, hTau/mTau^+/−^ and hTau/mTau^−/−^, mice were challenged with LPS (100, 250, or 330 μg/kg, *i.v*.) or its vehicle PBS. Their brains were collected 4 h later, immediately after behavioral assessment, for measurement of phosphorylated tau at the pS202 and pS396/404 epitopes and total tau (tau 46), by western immunoblotting **(A)**. In the absence of immune stimulation, expression of 4R mTau in hTau mice (hTau/mTau^+/−^) exacerbated pS202 **(B)** and pS396/404 **(C)** tau phosphorylation. Pairwise comparisons: ^##^*p* < 0.01; ^###^*p* < 0.0001 vs. hTau/mTau^−/−^ mice. In response to LPS, tau dephosphorylation occurred in hTau/mTau^+/−^, regardless of the dose, at both epitopes **(B,C)**, while in hTau/mTau^−/−^, the 100 μg/kg dose was selectively effective in reducing pS202 levels **(B)**, but total tau levels were unaltered by LPS **(D)**. This indicates that increasing 4R tau in hTau mice increased their tau phosphorylation potential at baseline as well as tau dephosphorylation potential in response to immune stimulation. Data are expressed as means ± SEM. *n* = 7–9/group. Pairwise comparisons: *p < 0.05; **p < 0.01; ***p < 0.0001 vs. PBS.

**Figure 4:**
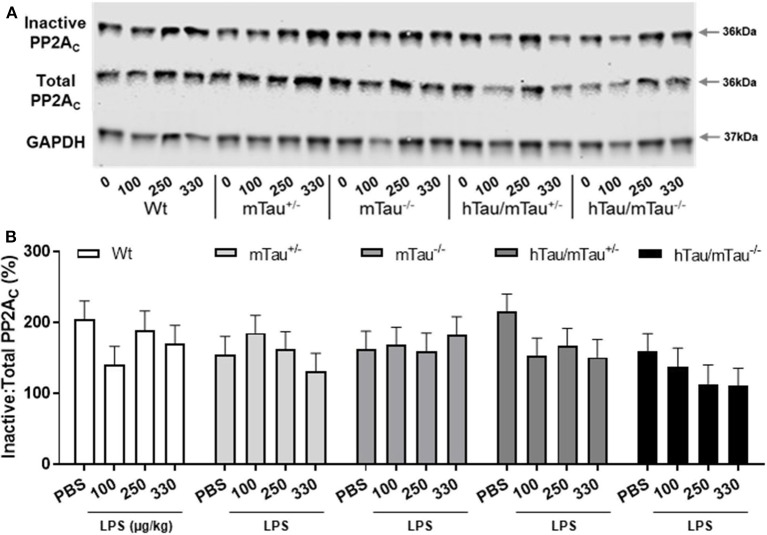
LPS failed to significantly increase protein phosphatase 2A catalytic (PP2A) activity. Three-month-old male Wt, mTau^+/−^, mTau^−/−^, hTau/mTau^+/−^, and hTau/mTau^−/−^ mice were challenged with LPS (100, 250, or 330 μg/kg, *i.v*.) or its vehicle PBS. Their brains were collected 4 h later, immediately after behavioral assessment. Since we found that LPS was able to dephosphorylate tau in both hTau models, we also assessed activity of the main tau phosphatase in the brain (PP2Ac) by western immunoblotting of inactive and total PP2Ac levels **(A)**. PP2A activity was estimated by the ratio of inactive: total PP2Ac **(B)**, whereby a reduction in this ratio would indicate increase PP2A activity. Although this ratio tended to be lower in LPS-treated hTau mice, regardless of the mTau phenotype, this failed to reach statistical significance. Data are expressed as means ± SEM. *n* = 7–9/group.

Next, we determined whether low doses of LPS affect hippocampal tau phosphorylation and the extent to which this can be modulated by 4R tau availability in hTau mice. We used western immunoblotting to quantify the levels of tau phosphorylation epitopes pS202 and pS396/404, which are predominantly associated with early-stage (18) and late-stage (62) tau pathology, respectively. They have been found to be induced by septic doses of LPS in tau models *in vivo* (35, 37, 63). In contrast, we found, 4 h after inoculation with systemic LPS, reduced hippocampal levels of pS202 and pS396/404 in hTau/mTau^+/−^ mice regardless of the dose (*p* < 0.05 vs. PBS-treated hTau/mTau^+/−^ mice in all cases, Figures 3B,C), while in hTau/mTau^−/−^ mice, LPS selectively lowered tau phosphorylation at the pS202 epitope at the lowest (100 μg/kg) dose (*p* < 0.05 vs. PBS-treated hTau/mTau^−/−^ mice, Figure 3B). In hTau/mTau^+/−^, LPS also significantly lowered the pS202:tau46 ratio at the 100 and 250 μg/kg doses (*p* = 0.008 and *p* = 0.004 vs. PBS-treated mice, respectively, Supplementary Figure 4A), and pS396/404:tau46 ratio at the 100 μg/kg dose only (*p* = 0.04 vs. PBS-treated mice, Supplementary Figure 4B), but significant reductions in the phosphorylated: total tau ratios were particularly observed in Wt mice at all LPS doses (Supplementary Figures 4A,B), despite the lack of significant alterations in tau species (Figures 3A–D). Thus, the enhanced tau phosphorylation caused by increased 4R tau levels is associated with a greater response of hTau/mTau^+/−^ mice to low LPS doses. Total tau levels remained constant, regardless of the genotype (Figure 3D), suggesting that tau dephosphorylation is the likely mechanism underlying reduced tau phosphorylation following LPS administration. We therefore looked at whether LPS modulated the activity of PP2Ac, the most important tau phosphatase in the central nervous system (CNS), by quantifying inactive and total hippocampal PP2Ac levels by western immunoblotting. However, LPS failed to significantly impact PP2Ac activity at 4 h in all genotypes, although there was a trend for a selective increase in PP2Ac activity in hTau/mTau^+/−^ mice, suggested by the non-significant reduction in the ratio of inactive to total PP2Ac seen with all three LPS doses (Figure 4B). Furthermore, LPS, regardless of the dose, failed to induce tau aggregation in all genotypes, as evidenced by the absence of sarkosyl-insoluble total and pS396/404 tau in the whole brain of 3-month-old mice (Supplementary Figure 5A), where we confirmed the impact of 4R Tau availability on soluble levels of these two markers, seen in their hippocampi (Figures 1A,C,D). All LPS doses indeed lowered whole-brain levels of phosphorylated tau at the pS396/404 epitope in hTau/mTau^+/−^ (all *p* < 0.0002 vs. PBS-treated hTau/mTau^+/−^), but not hTau/mTau^+/−^, mice (Supplementary Figure 5C) without affecting total tau levels (Supplementary Figure 5D).

### LPS-Induced Tau Dephosphorylation Persists for at Least 24 h in hTau/mTau^+/−^ Mice

**Figure 5:**
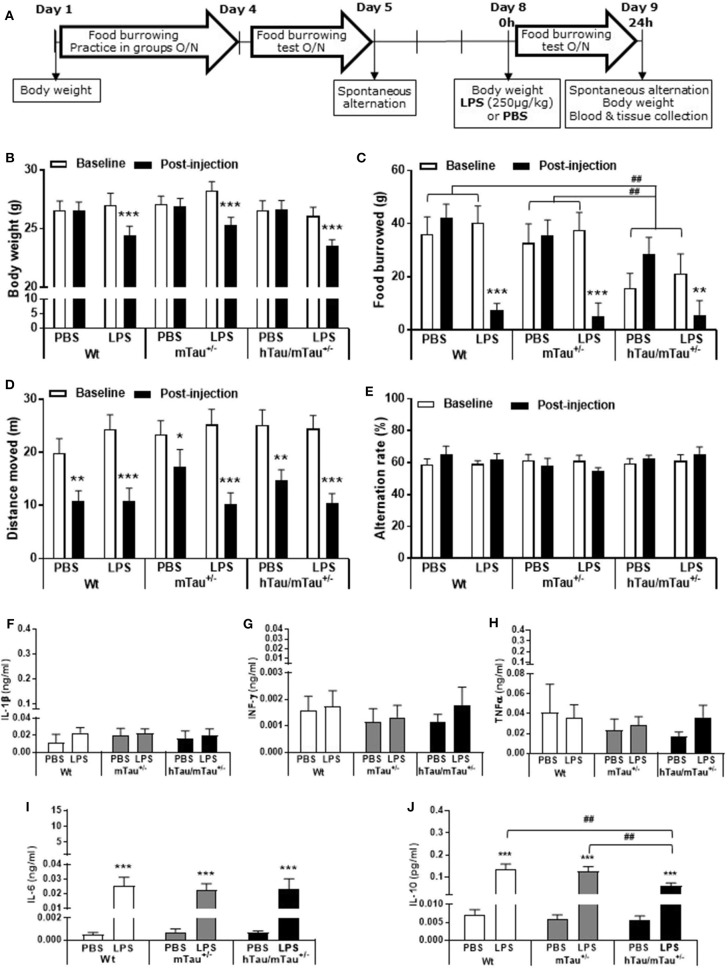
Persistence of systemic LPS effects in hTau/mTau^+/−^ mice at 24 h post-injection. Data are expressed as means ± SEM. **(A)** Timeline of the experiment. Three-month-old male Wt, mTau^+/−^ and hTau/mTau^−/−^ mice were subjected to baseline assessment of food burrowing and spontaneous alternation performance prior to receiving a tail vein injection of LPS (250 μg/kg) or its vehicle (PBS; *n* = 9–10/group). Induced sickness effects were tested at 24 h post-injection by measuring body weight loss, exploratory drive in the spontaneous alternation test, and food burrowing behavior prior to blood and tissue collection. Sickness symptoms were still present 24 h after LPS administration, as reflected by the body weight loss **(B)** and suppression of food burrowing behavior **(C)** seen in LPS-treated mice, regardless of the genotype. The reduction in performance seen in PBS-treated mice indicated that exploratory drive in the spontaneous alternation test was affected by repeated testing, but this was more pronounced in LPS-treated mice **(D)**, also indicating residual sickness effects; however, spontaneous alternation performance did not differ from baseline levels **(E)**. **(F–J)** LPS-induced circulating cytokines. Upper segment corresponds to the range of induced levels measured 4 h after inoculation with LPS. Indicating a resolution of the pro-inflammatory cytokine response to LPS, a return to baseline values was observed at the 24 h time point for IL-1β **(F)**, IFN-γ **(G)**, and TNFα **(H)**. Circulating levels of IL-6, known to exert anti-inflammatory effects at a low dose, remained elevated in all genotypes but to a much lower extent than seen at the 4 h time point **(I)**. Concentrations of the anti-inflammatory cytokine IL-10 were also still elevated but at levels close to those measured at 4 h post-LPS and to a lower extent in hTau/mTau^−/−^ mice than in Wt and mTau^+/−^ mice **(J)**. This indicates than sickness symptoms persist at 24 h after LPS administration, despite the resolution of the systemic pro-inflammatory cytokine response. We also replicated the characteristic food burrowing deficit of hTau mice in the absence of immune stimulation **(C)**. Pairwise comparisons: *p < 0.05, **p < 0.01, ***p < 0.0001 vs. PBS or baseline; ^##^*p* < 0.01 vs. other genotypes. *n* = 9–10 per group.

**Figure 6:**
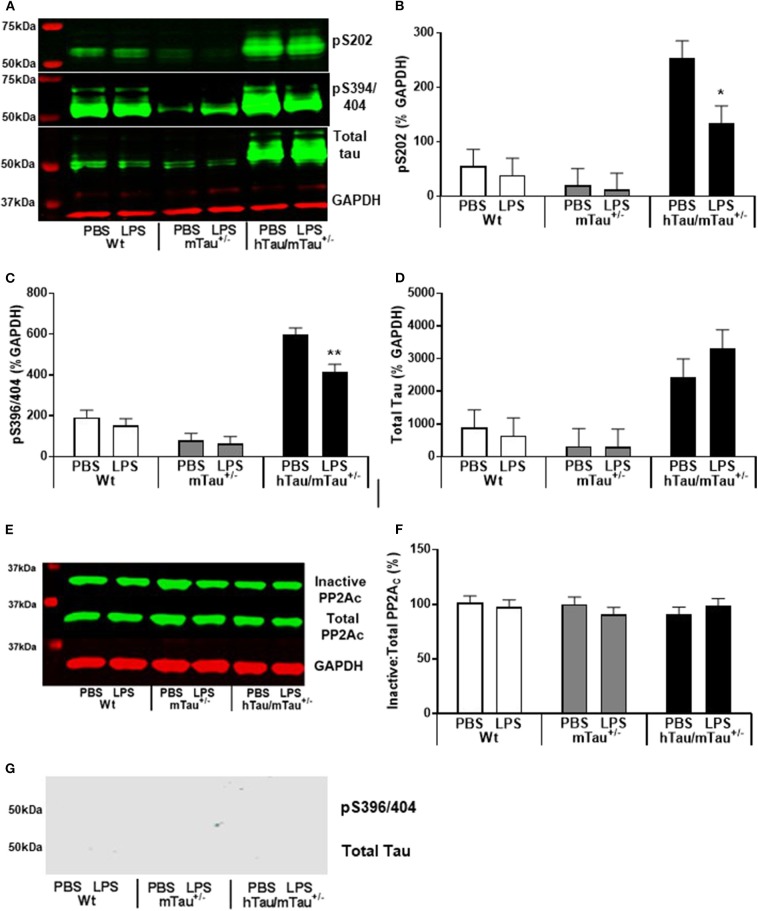
Persistence of tau dephosphorylating effects of systemic LPS in hTau/mTau^+/−^ mice at 24 h post-injection. Three-month-old male Wt, mTau^+/−^, and hTau/mTau^−/−^ mice were challenged with LPS (250 μg/kg, *i.v*.) or its vehicle PBS. Their brains were collected 24 h later, immediately after assessment of sickness symptoms for the determination by western immunoblotting of hippocampal soluble phosphorylated tau at the pS202 and pS396/404 epitopes and total tau (tau 46) levels **(A)**, PP2A activity **(E)**, and whole brain insoluble total and phosphorylated tau **(G)**. pS202 **(B)** and pS396/404 **(C)** tau dephosphorylation persisted for at least 24 h after LPS administration, while levels of total tau **(D)** and PP2A activity **(F)** remained unaltered. There was also no evidence of insoluble tau **(G)**. Data are expressed as means ± SEM. Pairwise comparisons: *p < 0.05; **p < 0.01 vs. PBS.

All together, the data reported above suggest that reducing the predominance of 3R tau by increasing 4R tau availability exacerbates tau hyperphosphorylation in 3-month-old hTau mice, as well as the tau dephosphorylating effect of low LPS doses, 4 h after inoculation. We therefore next investigated in hTau/mTau^+/−^ mice the persistence of LPS effects at the 250 μg/kg dose (Figure 5A), which produced robust pro-inflammatory response and tau dephosphorylation. We chose a 24 h time point, which is expected to correspond to the resolution of the pro-inflammatory cytokine response following acute LPS administration in mice (64). We found that LPS-treated hTau/mTau^+/−^ mice and their LPS-treated Wt and mTau^+/−^ littermates were still experiencing LPS-induced sickness, manifested by a significant weight loss in all genotypes [~ 10%; *p* < 0.0001 vs. baseline pre-injection weight in all cases; treatment × time: *F*_(1, 53)_ = 163.89, *p* < 0.0001; Figure 5B], as well as suppression of food burrowing behavior [*p* < 0.03 vs. baseline pre-injection performance in all cases; treatment × time: *F*_(1, 53)_ = 41.16, *p* < 0.0001; Figure 5C]. However, while the injection and/or repeated testing attenuated spontaneous exploration of the Y-maze during the spontaneous alternation test [time: *F*_(1, 52)_ = 122.07, *p* < 0.0001; Figure 5D], the reduction in the distance moved in the apparatus was also greater in LPS-treated than PBS-treated mice [treatment × time: *F*_(1, 52)_ = 8.07, *p* = 0.006; ~ −23–29% in PBS-treated mice, all *p* < 0.03 vs. baseline performance; and −52–61% in LPS-treated mice, all *p* < 0.0001 vs. baseline performance; Figure 5D], indicating residual sickness effects of the immune challenge. Spontaneous alternation performance, a measure of spatial working memory, was not affected (Figure 5E). Although circulating levels of the pro-inflammatory cytokines IL-1β, INFγ, and TNFα had returned to baseline values in all three genotypes (Figures 5F–H), the pro-inflammatory cytokine IL-6, known to exert anti-inflammatory effects at low doses (65, 66), remained elevated in all three genotypes 24 h after systemic LPS administration [treatment: *F*_(1, 51)_ = 45.16, *p* < 0.0001], albeit to a much lesser extent than observed at the 4 h time point (Figure 5I). Concentrations of the anti-inflammatory cytokine IL-10 remained elevated in all three genotypes [treatment: *F*_(1, 51)_ = 76.34, *p* < 0.0001] at levels closed to those measured at the 4 h time point, but to a lower extent in LPS-treated hTau/mTau^+/−^ mice than in LPS-treated Wt (*p* < 0.0009) and mTau^+/−^ (*p* < 0.0018) mice [genotype × treatment: *F*_(2, 51)_ = 3.93, *p* = 0.026; Figure 5J]. Furthermore, at 24 h post-injection, hippocampal tau dephosphorylation was still seen in LPS-treated hTau/mTau^+/−^ mice at both the pS202 (*p* = 0.01 vs. PBS-treated mice, Figures 6A,B) and pS396/404 (*p* = 0.04 vs. PBS-treated mice, Figures 6A,C) epitopes, while total levels of tau (Figures 6A,D) and PP2Ac activity (Figures 6E,F) remained unaltered. We again assessed sarkosylinsoluble tau at 24 h post-inoculation with LPS, and there was no evidence of either total or pS396/404 tau in sarkosyl fractions, regardless of treatment (Figure 6G), indicating that LPS did not induce tau aggregation (Figure 6F).

## Discussion

To investigate whether reducing the predominance of the toxic 3R Tau isoform affects susceptibility to systemic inflammation and LPS-induced changes in tau phosphorylation, we increased 4R tau availability in hTau mice by breeding them on a heterozygous mTau knockout background, as adult mTau consists solely of 4R tau (67). We found in 3-month-old mice that increasing 4R availability in hTau mice exacerbated tau hyperphosphorylation but not total tau in the absence of immune stimulation as well as tau dephosphorylation in response to all three doses of LPS tested. The increased phosphorylation potential at baseline was not accompanied by differences in early behavioral and physiological disturbances characteristic of hTau mice, but the greater dephosphorylation potential of hTau/mTau^+/−^ under immune stimulation was associated with a stronger acute behavioral sickness at the lowest (100 μg/kg dose) and milder sickness and cytokine responses to the highest (330 μg/kg) LPS dose.

The predominance of 3R tau has been linked to the pathogenesis of tauopathies, correlating with the severity of NFT pathology in the post-mortem AD brain and driving pathological changes in hTau mice. Based on recent reports showing that increasing 4R tau levels without changes in 3R tau prevented the development of cognitive deficits in these mice, as well as the accumulation of insoluble and phosphorylated tau, without altering total tau levels (46), we predicted a milder tau pathology and behavioral phenotype in hTau/mTau^+/−^ than in hTau/mTau^−/−^. In contrast, we found that the higher abundance of the 4R tau isoform selectively increased tau phosphorylation in 3-month-old hTau mice, although the associated increases in phosphorylated: total tau ratios failed to reach statistical significance. This exacerbation of pathological tau could be resulting from differences in the extent to which we altered the isoform ratio in the model. Espindola et al. (46) indeed reprogrammed 3R to 4R tau isoforms to a ratio of ~1 to mimic the balance seen in the healthy human brain, while with our breeding strategy, 3R tau remained the predominant isoform. While a balance between the two isoforms may be critical to protect from the development of tau pathology, both 3R and 4R tau were found toxic in drosophila, with isoform-specific mechanisms of tau toxicity and phosphorylation potential (68). There is also evidence to suggest that 4R tau is toxic in the mouse brain. Indeed, the splicing of 3R−4R tau in hTau mice, leading to an isoform ratio favoring 4R tau by increasing 4R tau and correspondingly decreasing 3R tau without affecting total tau levels, was also found to exacerbate tau phosphorylation and behavioral abnormalities (47). Differences in structure and function between human and mTau have been reported, but it is unlikely that this contributes to the tau dephosphorylating effects of increased 4R availability seen in response to LPS. mTau mice indeed lack residues 17–28, but *in vitro* studies using brain extracts from AD patients have found that this particularly affected the binding of mTau to proteins regulating energetic processes in neurons (69). And while several studies have shown that the presence of mTau that is predominantly 4R delays the pathological accumulation of human tau, with exacerbated disease progression being observed in tau models after full removal of mTau [reviewed in (70)], we found here that keeping 50% of mTau exacerbated tau hyperphosphorylation in hTau mice in the absence of immune stimulation, although this was protective in response to LPS. Furthermore, in the 3xTg model of AD, endogenous mouse tau was found to be hyperphosphorylated and to significantly co-aggregate with human tau, while the deletion of the mTau gene in this model lowered soluble total and phosphorylated tau, as well as insoluble total tau, without impacting on cognition (70). This suggests that the ability of 4R mTau to modulate pathogenic features of human tau in mouse models is not due to species differences, but a mechanistic study comparing the impact of 4R tau from both species would be needed to confirm this point. We previously reported that food burrowing is the most prominent early behavioral deficit in hTau mice, regardless of the mTau background (48, 49). As seen in the 3xTg model, we found here that the expression of 4R mTau in hTau mice did not worsen the food burrowing impairment, nor the lower body mass, despite the increased tau phosphorylation. Whether or not this has also an impact on tau aggregation could not be investigated here, as we, unexpectedly, did not find evidence of insoluble total and/or phosphorylated tau at the ages investigated. As we discussed before (49), the C57BL/6J background was found to weaken the phenotype of a number of disease models and could explain the milder phenotype of hTau mice obtained from The Jackson Laboratory, as compared to that originally described (36) on a mixed Swiss Webster/129/SvJae/C57BL/6 background.

Altered immune status is a common feature of tauopathies (71, 72), regardless of their classification in terms of 3R/4R predominance. To the best of our knowledge, no study has investigated whether specific tau isoforms differentially modulate immune responses. 3R and 4R tau were reported to differ in their physiological function and pathological role (68), and interplays between tau mechanisms and immune responses are thought to contribute to tau pathogenesis (71, 72). We therefore hypothesized that increasing the abundance of 4R tau would impact on tau pathology as well as on behavioral and physiological responses to mild systemic inflammation induced by LPS. We found here that the expression of 4R murine in hTau mice had little impact on LPS-induced sickness and circulating cytokine levels at 4 h post-inoculation, a time point corresponding to the peak cytokine response (73–75), but this may be dose dependent. Indeed, while behavioral suppression increased in severity with raising doses of LPS in Wt, hTau/mTau^−/−^ mice and their mTau knockout littermates, it was more severe in hTau/mTau^+/−^ mice and heterozygous mTau knockout littermates at the lowest dose used (100 μg/kg) but less severe at the highest dose (330 μg/kg). In hTau/mTau^+/−^ mice, the latter was associated with reduced secretion of pro-inflammatory cytokine INFγ and anti-inflammatory cytokine IL-10. This suggests that while increasing availability of 4R Tau in hTau mice enhanced the sickness response to the lowest low LPS doses, it may limit the systemic impact of stronger pro-inflammatory stimuli. LPS-induced tau dephosphorylation was also more robust in hTau mice expressing 4R mTau, occurring at all three doses on both pS202 and pS396/404 epitopes and persisting for at least 24 h after inoculation with LPS.

While LPS was generally found to exacerbate tau phosphorylation in models of tauopathies (35, 37, 63), our data argue in favor of a protective effect of systemic LPS on tau pathology. The strength of the immune stimulation is likely to be a factor, as the above studies used either LPS doses more akin to sepsis or a chronic dosing regimen. Consistent with our data, recent studies also suggest that mild systemic inflammation may be beneficial to tau pathology. A single 150 μg/kg LPS challenge was found to decrease the levels of aggregated tau through induction of the autophagy pathway in the P301S model (76). Furthermore, collagen-induced arthritis in the 3xTg model was reported to decrease tau phosphorylation and the number of neurons affected by tau aggregates (77). As this arthritis model is associated with chronic systemic inflammation that is truly mild (78, 79), this also suggests that systemic inflammation may not have a detrimental effect on tau pathology in AD. Supporting a potential dose dependency in the occurrence of protective *vs*. detrimental effects of LPS on tau pathology, tau dephosphorylation occurred only at the pS202 epitope and only at the lowest (100 μg/kg) dose in hTau/mTau^−/−^ mice, while in hTau/mTau^+/−^ mice, the highest (330 μg/kg) LPS dose was the least effective in reducing both pS202 and pS396/404 levels.

The mechanisms whereby LPS limits tau hyperphosphorylation could not be identified in the present study. It could result from either decreased kinase activation or increased phosphatase activation. We hypothesized that alterations in phosphatase activity were the source for tau dephosphorylation in the current study, with PP2A the likely culprit. PP2A accounts for about 1% of cellular content within the brain and is the predominant phosphatase that affects tau phosphorylation (80). LPS has been shown to induce PP2A activation *in vitro* in endothelial cells (81, 82). Furthermore, LPS and superoxide, one of the downstream products of LPS-induced inflammation (83), have been shown to induce PP2A activation in hippocampal slices (84, 85), indicating that LPS can directly and indirectly induce PP2A activation within the CNS. To assess PP2A activity in the current study, western blot analyses of the PP2A catalytic subunit were conducted after incubation with or without NaOH, giving a measure of inactive:total PP2Ac. Despite the lack of significant alterations in this measure, there was a trend for a decrease in both hTau genotypes 4 h after being challenged with LPS. A more sensitive assay would be needed to categorically ascertain whether changes in PP2A activity were associated with LPS-induced tau dephosphorylation. Various kinases have been implicated in the exacerbation of tau phosphorylation by high LPS doses but with discrepancies between studies (39). Increased activity of cyclin-dependent kinase 5 and/or glycogen synthase kinase-3β was most commonly found associated with LPS-induced tau hyperphosphorylation in Wt and 3xTg mice (37, 38), but increased autophagic degradation rather than reduced kinase activity was found associated with the reduced tau pathology caused by a low 150 μg/kg LPS dose in P301S mice (76). Their implication in the modulation of LPS-induced tau dephosphorylation by 4R tau will need to be investigated in future studies.

## Conclusions

While previous studies have found that a full mTau background prevents the development of tau pathology in the hTau model (36), we found here that reducing mTau expression by half exacerbates tau phosphorylation in this model, suggesting that the effect of 4R mTau on pathogenesis in hTau mice is dose dependent. Our data also further demonstrate that modulation of the 4R:3R tau isoform ratio effectively modulates disease severity in this model. However, we are the first to show that this also affects the impact of systemic inflammation on tau phosphorylation, behavioral sickness, and circulating cytokine levels. Despite leading to an increase in tau phosphorylation potential in the absence of immune stimulation, elevated 4R tau levels in hTau mice were also associated with an increased tau dephosphorylation potential in response to LPS, but the underlying mechanism of this needs to be investigated in future studies. The attenuated systemic response of hTau/mTau^+/−^ mice to the highest LPS dose tested also suggests that 4R tau may actually offer some protection against disease-exacerbating effects of systemic inflammation.

## Data Availability Statement

The raw data supporting the conclusions of this article will be made available on request to any qualified researcher.

## Ethics Statement

The animal study was reviewed and approved by the University of Nottingham Animal Welfare and Ethical Review Board (AWERB).

## Author Contributions

MB, JG, LD, PA, and M-CP contributed to the conception and design of the study. MB performed the experiments. MB and M-CP performed the statistical analysis and wrote the manuscript. All authors contributed, read and approved the submitted version.

### Conflict of Interest

JG and PA are employees of Eisai Ltd. LD is an employee of Cerevance Ltd. The remaining authors declare that the research was conducted in the absence of any commercial or financial relationships that could be construed as a potential conflict of interest.
