# Supplementary material for: Increasing Tau 4R Tau Levels Exacerbates Hippocampal Tau Hyperphosphorylation in the hTau Model of Tauopathy but Also Tau Dephosphorylation Following Acute Systemic Inflammation
Source: Front Immunol. 2020 Mar 5;11:293. doi: 10.3389/fimmu.2020.00293 (PMC7066213; doi:10.3389/fimmu.2020.00293)
Supplement: Supplementary file 1 [file Data_Sheet_1.PDF]

Supplementary Material

Increasing tau 4R tau levels exacerbates hippocampal tau hyperphosphorylation in the hTau model of tauopathy but also tau dephosphorylation following acute systemic inflammation.

Matthew Barron, Jane Gartlon, Lee A. Dawson, Peter J. Atkinson and Marie-Christine Pardon

1 Supplementary Figures

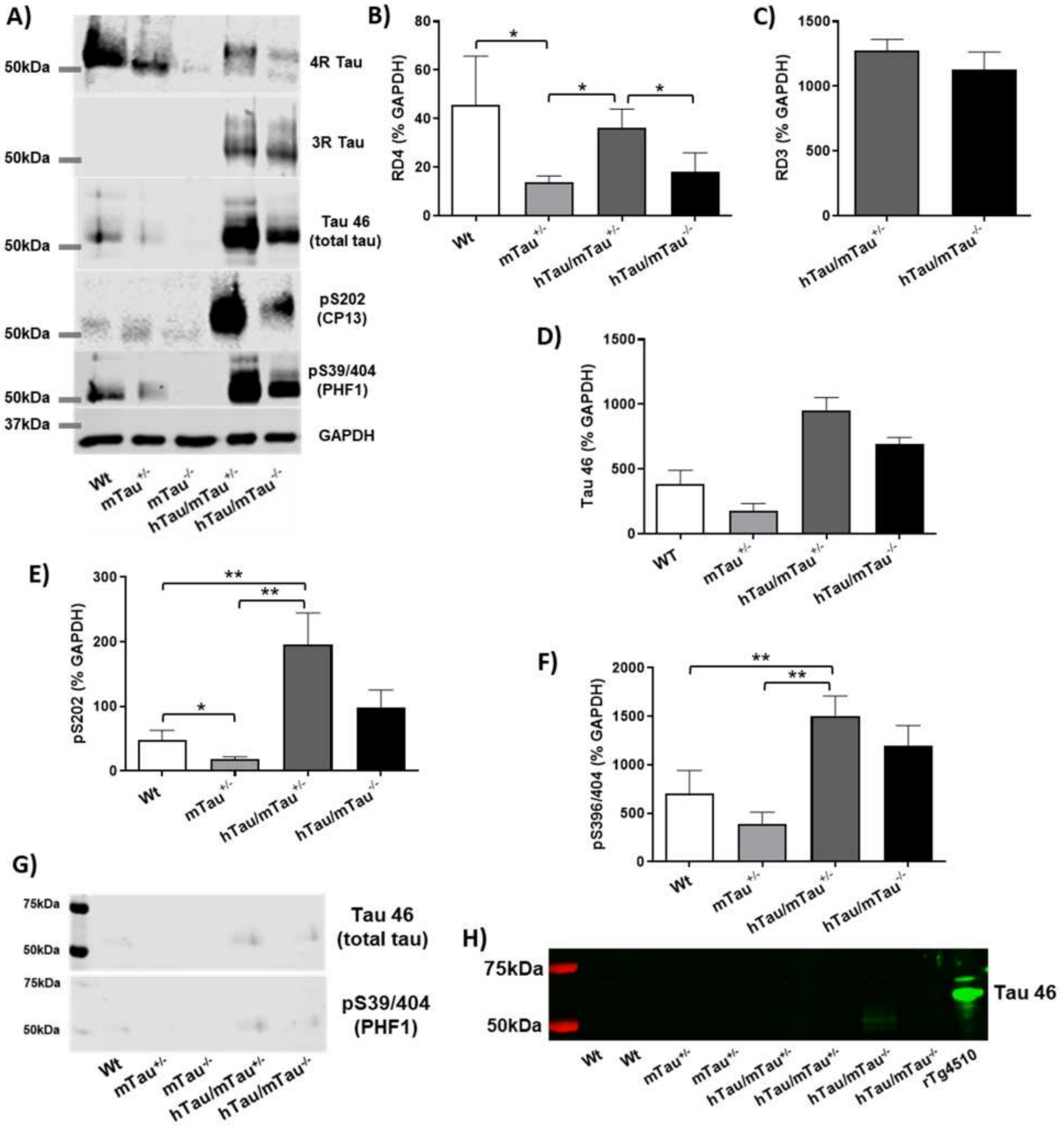

Supplementary Figure 1. Whole brain tau levels in soluble (A-F) and Sarkosyl-insoluble (G) fractions from 9-month-old Wt, $mTau^{+/-}$, $hTau/mTau^{+/-}$ and $hTau/mTau^{-/-}$ mouse brains. Representative western-immunoblotting of tau species, showing the absence of tau in full knockout $mTau^{-/-}$ mice (A). As expected, hTau mice bred on a heterozygous murine tau knockout background ($hTau/mTau^{+/-}$), presented with elevated 4R (B) but not 3R (C) tau compared to hTau mice bred on a full mTau knockout ($hTau/mTau^{-/-}$) background. This was associated with elevated levels of total (D) and phosphorylated (E-F) tau. Both 9-month-old $hTau/mTau^{+/-}$ and $hTau/mTau^{-/-}$ mice failed to exhibit aggregated tau (G). Validation of tau aggregation assay using the rTg4510 model of tauopathy. Data were rank-transformed for statistical analyses but are expressed as Means $\pm$ SEM non-normalised responses. Pairwise comparisons following one-way ANOVA: * $p<0.05$; ** $p<0.01$, *** $p<0.0001$. n=3-4 mice per group.

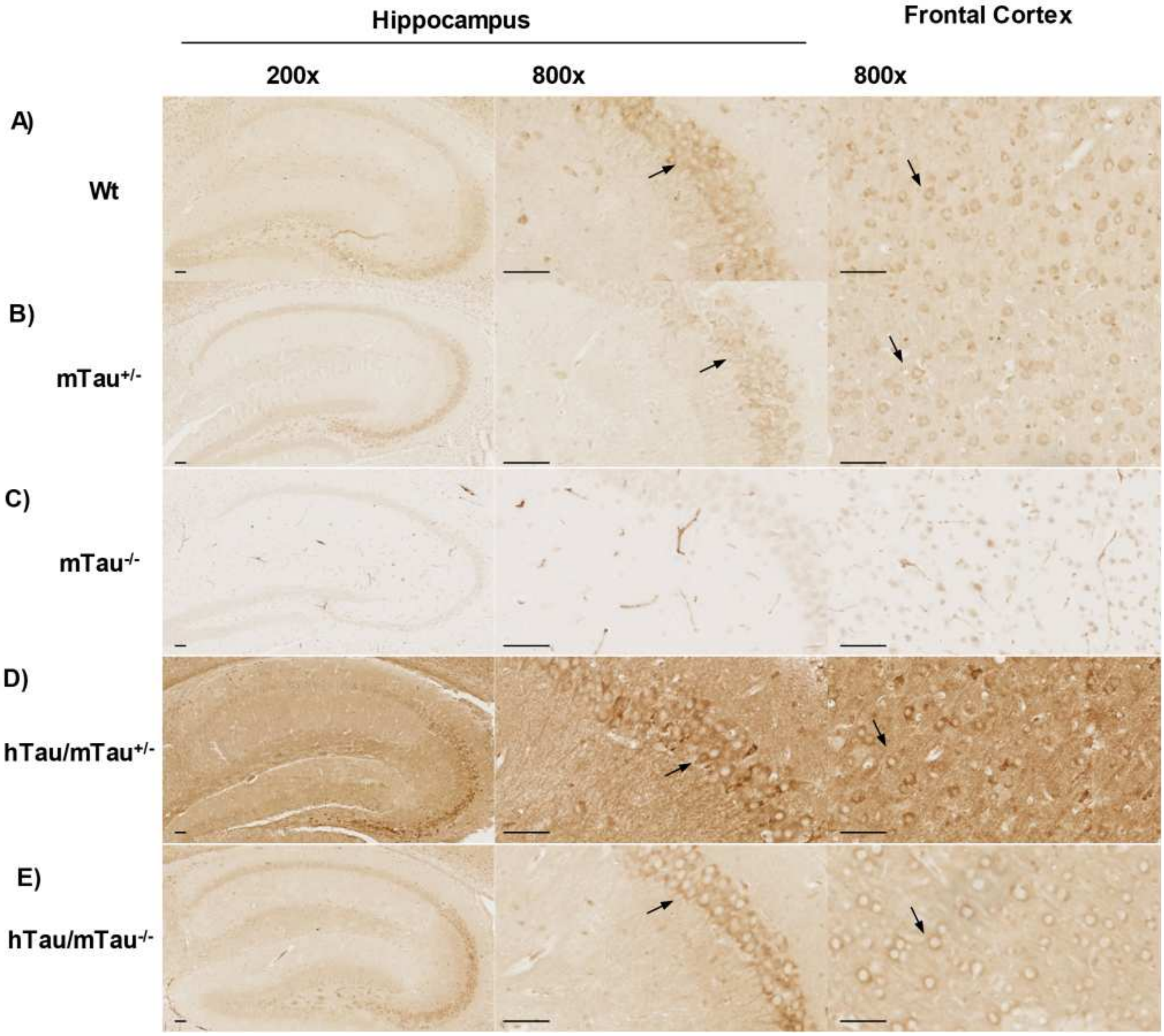

Supplementary Figure 2: Hippocampal phosphorylated tau localisation in 3-month-old male hTau mice bred on either a full or heterozygous murine tau knockout background. Brain slices were stained for pS202, revealing strong CP13 positive rings around hippocampal and cortical cell bodies of both hTau/mTau$^{+/-}$ (D) and hTau/mTau$^{-/-}$ (E). There was mild evidence for the presence of some pS202 tau in the somatodendritic compartment of Wt (A) and mTau$^{+/-}$ (B) mice, but no positive staining was observed in mTau$^{-/-}$ mice (C). This indicates a high concentration of somatodendritic phosphorylated tau and evidence of tau redistribution in both hTau models at 3 months of age. Black arrows indicate cells with a somatodendritic location of tau. Scale bar: 50 µm.

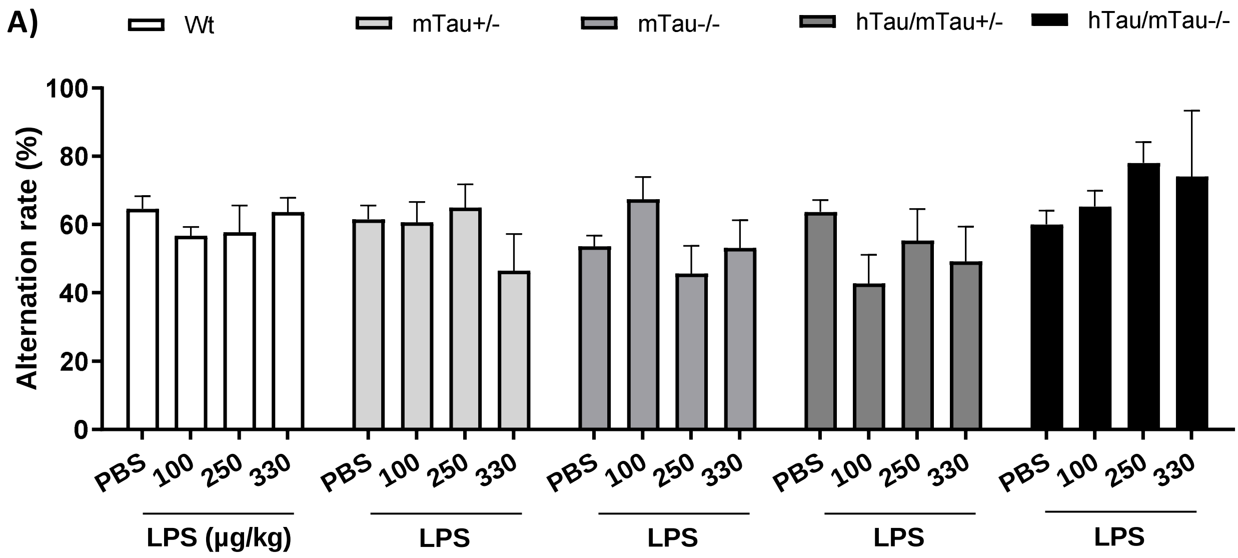

Supplementary Figure 3: Three-month-old Wt, mTau$^{+/-}$, mTau$^{-/-}$, hTau/mTau$^{+/-}$ and hTau/mTau$^{-/-}$ mice were assessed for spatial working memory performance in the spontaneous alternation test 4 hours after being challenged with PBS or LPS at a dose of 100, 250 or 330 µg/kg i.v. While there was an overall effect of genotype ($F_{(4,140)} = 3.64$, $p = 0.007$), regardless of treatment, pairwise comparisons did not reveal any differences in performance between genotypes in PBS-treated mice. LPS did not significantly alter performance in any genotype (LPS doses: $F_{(3,140)} = 0.58$, $p = 0.63$; Genotype X LPS doses: $F_{(12,140)} = 1.51$, $p = 0.13$). Two-Way ANOVA with arm entries as a covariate, to control for confounding effects of LPS-induced behavioural suppression on spontaneous alternation behaviour. Mice who did enter more than one arm due to suppressive effects of LPS were excluded as their spontaneous alternation rate could not be calculated. n=5-9 per group.

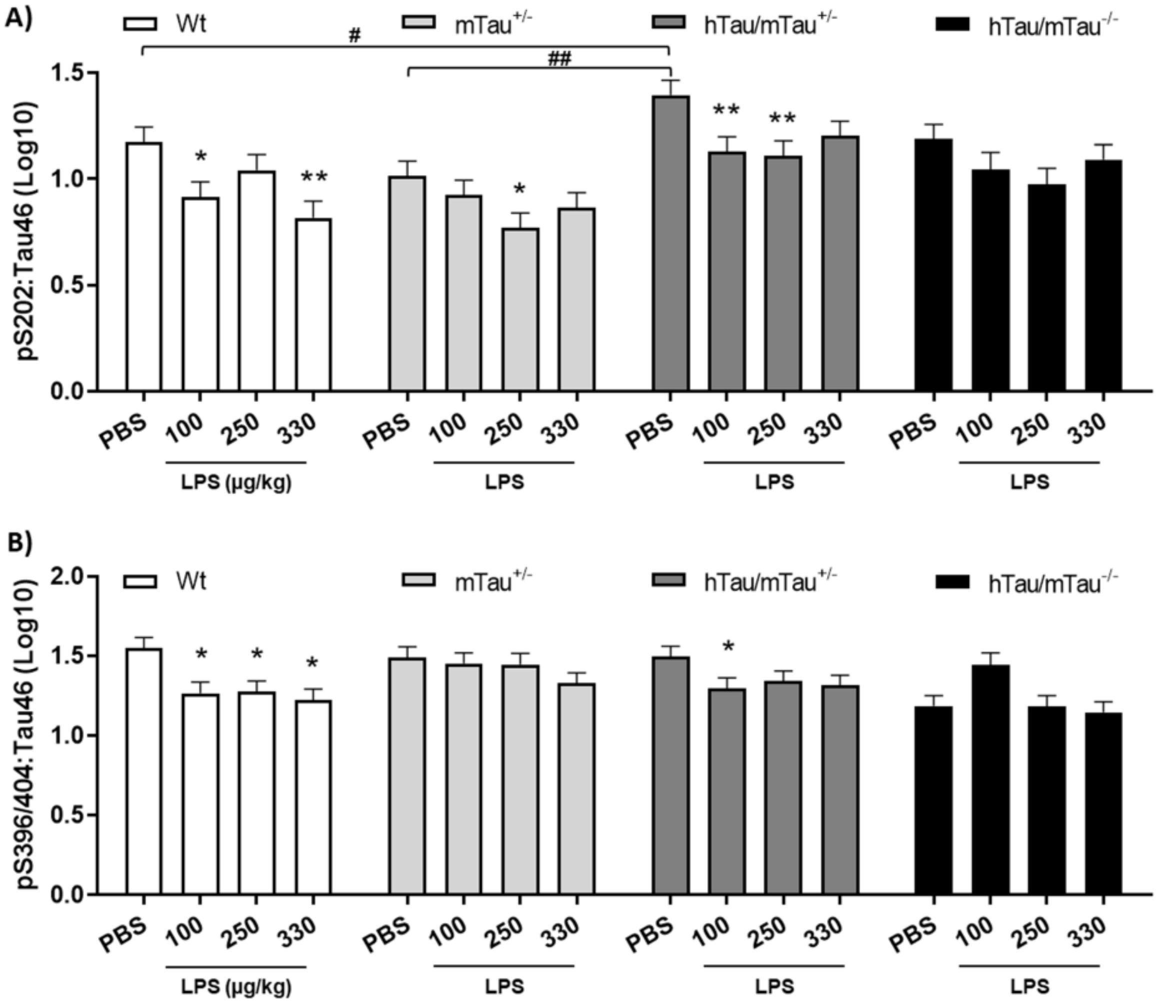

Supplementary Figure 4: Ratios of phosphorylated tau species over total tau in the hippocampi of 3-month-old Wt, mTau$^{+/-}$, hTau/mTau$^{+/-}$ and hTau/mTau$^{-/-}$ mice, 4 hours after injection with LPS (100, 250, or 330 µg/kg, *i.v.*) or its vehicle PBS. Early pathological stage phosphorylated tau at the pS202 epitope over tau46 (A) and late pathological stage phosphorylated tau at the pS396/404 epitope over tau46 (B). The major reduction in the levels of phosphorylated tau at both epitopes induced by all doses of LPS in hTau/mTau$^{+/-}$ was associated with a significant reduction in the ratio of phosphorylated over total tau at the lowest doses: A) 100 and 250 µg/kg for pS202 and B) 100 µg/kg for pS396/404. Although LPS had no significant effect on either tau species in Wt and mTau$^{+/-}$ mice, it significantly lowered the ratio of both phosphorylated tau species over total tau in Wt mice (A&B), and of pS202 over tau46, at the 250 µg/kg dose only, in mTau$^{+/-}$. Each of the three tau species were quantified on separate blots, but co-incubated with GAPDH as a loading control. The resulting data was normalised to GAPDH prior to calculation of the ratio of phosphorylated over total tau levels. Data are expressed as Means $\pm$ SEM and were Log10 transformed to normalise the distribution. Main effects in ANOVAs: pS202:tau46 (Genotype: $F_{(3,113)} = 14.5$, $p < 0.0001$; Treatment: $F_{(3,113)} = 6.92$, $p = 0.0003$) and pS396/404:tau46 (Treatment: $F_{(3,113)} = 5.65$, $p = 0.0012$). Pairwise comparisons following ANOVA: * $p<0.05$; ** $p<0.01$ *vs* PBS and $^{\#}$ $p<0.01$, $^{\#\#}$ $p<0.01$ *vs* Wt mice. n=7-9/group.

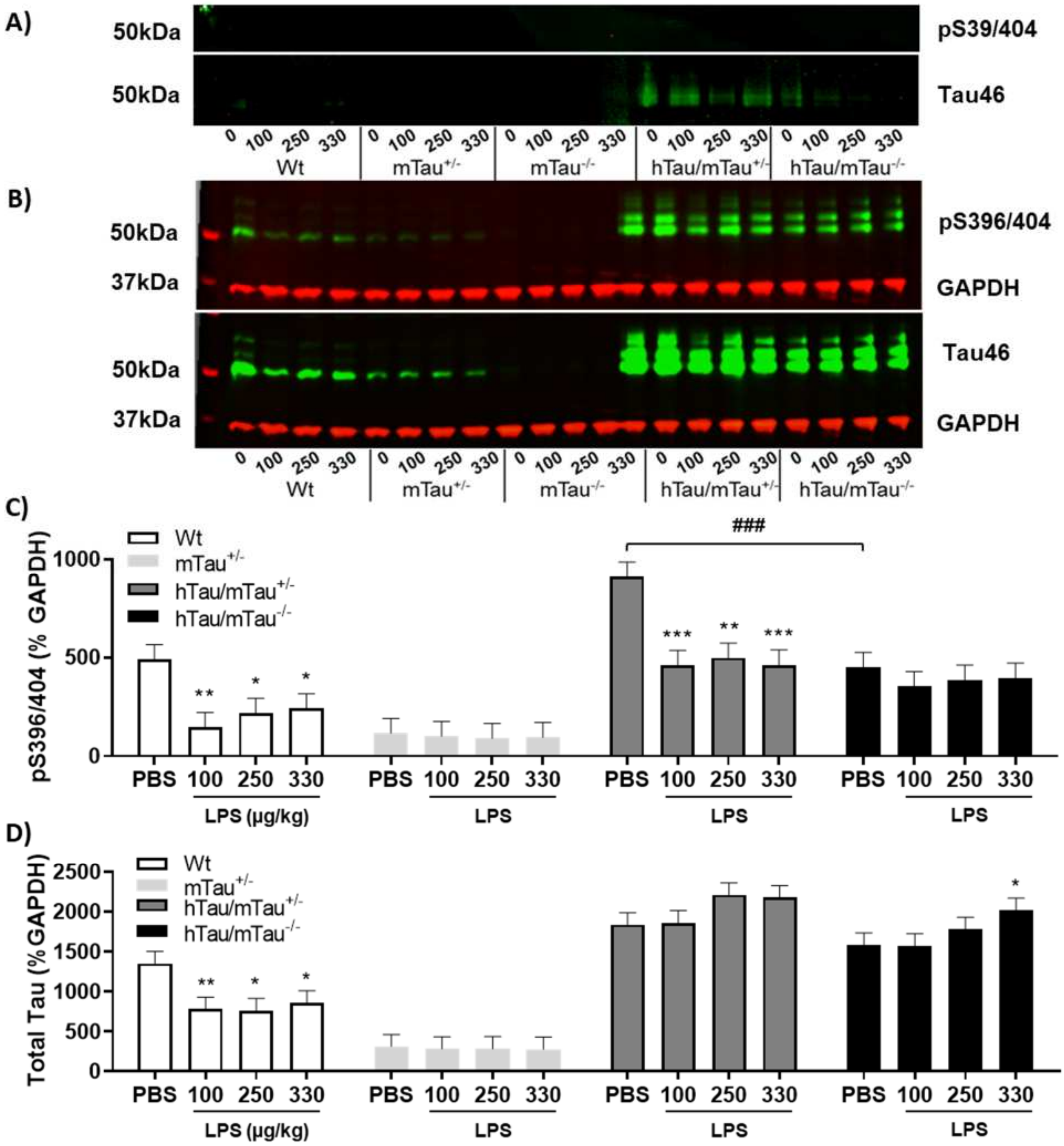

Supplementary Figure 5: Whole brain phosphorylated and total tau levels in Sarkosyl-insoluble (A) and soluble fractions (B-D) of 3-month-old Wt, mTau$^{+/-}$, hTau/mTau$^{+/-}$ and hTau/mTau$^{-/-}$ mice, 4 hours after injection with LPS (100, 250, or 330 µg/kg, *i.v.*) or its vehicle PBS. Representative western immunoblotting of tau species in the insoluble (A) and soluble (B) fractions confirming the absence of tau in mTau$^{-/-}$ mice. hTau mice bred on a heterozygous murine tau knockout background (hTau/mTau$^{+/-}$) exhibited higher levels of phosphorylated tau than hTau mice bred on a full mTau knockout (hTau/mTau$^{-/-}$) background (C), and greater tau dephosphorylation after LPS (C), consistent with hippocampal data. In contrast to hippocampal data, showing no effects of LPS in Wt mice, both phosphorylated (C) and total (D) tau levels were also reduced in the hemibrains of Wt mice. Data are expressed as Means $\pm$ SEM. Pairwise comparisons following ANOVA: * $p<0.05$; ** $p<0.01$, *** $p<0.0001$ vs PBS and $^{##}$ $p<0.01$ *vs* hTau/mTau$^{-/-}$ mice. n=6/group.
